# Cholinesterase research outreach project (CROP): point of care cholinesterase measurement in an Australian agricultural community

**DOI:** 10.1186/s12940-018-0374-1

**Published:** 2018-04-02

**Authors:** Jacqueline Cotton, John Edwards, Muhammad Aziz Rahman, Susan Brumby

**Affiliations:** 1 School of Medicine, Deakin University, 75 Pigdons Road, Waurn Ponds, VIC 3216 Australia; 2 National Centre for Farmer Health, Western District Health Service, Hamilton, VIC 3300 Australia; 3 School of Environment, Flinders University, Bedford Park, SA 5042 Australia; 4 Austin Clinical School of Nursing, La Trobe University, Heidelberg, VIC 3084 Australia

**Keywords:** Farmer, Organophosphate, Pesticides, Safety, Chemical, Cholinesterase

## Abstract

**Background:**

Australian farmers are routinely exposed to a wide variety of agrichemicals, including herbicides and insecticides. Organophosphate (OP) insecticides are widely used for agricultural production, horticulture and animal husbandry practices. Symptoms of OP toxicity are the results of inhibition of the enzyme acetylcholinesterase (AChE) which is found in many types of conducting tissue in human bodies such as nerve and muscle, central and peripheral tissues, motor and sensory fibres. Cholinesterase can be measured in red blood cells/erythrocytes (AChE) and plasma (PChE). This study aims to explore integration of AChE monitoring into routine health checks for those at risk and also to examine any association between AChE activity and agrichemical use in a Victorian farming community in Australia.

**Methods:**

This was a prospective cohort study, where farmers and non-famers were compared on the levels of AChE at four time points of baseline, 3–4 weeks, 6-weeks and at 9-weeks. Study participants (*N* = 55) were residents from South West Victoria, aged between 18 and 75 years, spoke English, and had not had a previous known acute chemical accident. A total of 41 farming (had been farming for more than 5 years) and a convenience sample of 14 non-farming individuals met the inclusion criteria. Testing of AChE was repeated for all participants with a maximum of three times over 10 weeks.

**Results:**

The integration of AChE monitoring was very well accepted by all participants. There was no significant difference in average AChE activity between farming and non-farming participants (one-way ANOVA *p* > 0.05) in this study. There was no significant difference between personal use of agricultural chemicals on farm and the levels of AChE at baseline (measurement 1) or any of the follow up periods (*p* > 0.05). However, the mean activity of AChE was significantly lower within follow up periods [F (2.633, 139.539) = 14.967, *p* < 0.001]. There was a significant reduction of AChE between the follow up at 3-weeks and 6-weeks period (*p* = 0.015).

**Conclusions:**

The routine monitoring of AChE may allow for early recognition of chronic low-level exposure to OPs when they are used by farmers, provided a reasonable estimate of baseline AChE is available. This work provides an evidence for recommending the integration of AChE monitoring into point of care (POC) procedures in rural health clinics and quantifying pesticide exposure and personal protection both on the farm and in the home. Farmer engagement is crucial to the successful integration of AChE monitoring into rural health clinics in Australia.

**Trial registration:**

ACTRN12613001256763.

## Background

Pesticides are substances that destroy, repel or attack pests that have a negative effect on productivity and profitability of a farming enterprise. Common pesticide groups include herbicides, insecticides, fungicides and rodenticides. Australian farmers and their workers are exposed to a wide variety of pesticides [1, 2]. Pesticide poisoning has been identified as an important occupational health problem in farmers and farm workers [3]. Organophosphate (OP) insecticides are widely used in agriculture. For example in animal husbandry practices (naphthalophos sheep drench),fruit and vegetable production (disulforon), crop and pasture production (dimethoate for insect pest control) and even in public health (malathion for human head lice). Organophosphates are regularly used in most farming operations and routes of human exposure include dermal absorption, inhalation and ingestion [4]. Australian farmers have increased their understanding of pesticide use and handling over the last two decades with the introduction of chemical user training. However, research suggests that despite this, many farmers and agricultural workers, do not meet the recommended personal protection equipment (PPE) standards for the handling and application of pesticides [5]. A recent study commissioned in 2015 by the Rural Industries Research and Development Corporation (RIRDC) reported that barriers to the current use of PPE (as reported by farmers) include the discomfort and availability for everyday use [6].

The OP class of insecticides affects the nervous system with reported acute poisoning most commonly occurring among agricultural workers and children [7, 8] in developing countries. Organophosphates have been associated with chronic neurological symptoms such as impaired memory, impaired fine motor skills control, OP-induced delayed neuropathy (OPIDN), and have been implicated in Parkinson’s disease [9]. The critical window for exposure to OP’s toxicants may occur years before the onset of neurological symptoms [9–11], and this is particularly relevant for sheep, crop and fruit and vegetable producers who may be exposed to long-term, low levels of OPs.

Western Victoria has long been a centre of sheep production in Australia, with a large number of farmers involved in and exposed to OP’s through routine sheep dipping (lice control), drenching (internal parasites) and jetting for blow fly control. Additional practices such as mixing contaminated clothing with household laundry and storing pesticides in the home are not only common causes of exposure to the farmers and farm workers, but also put other household members at risk, particularly children [12]. Work completed by the National Centre for Farmer Health (NCFH), Sustainable Farm Families™ [13] identified a strong interest from farm men and women who wish to investigate exposures and possible health impacts of agricultural chemical use.

Symptoms of OP toxicity are caused by inhibition of the enzyme acetylcholinesterase (AChE). Inhibition of AChE results in the subsequent accumulation of acetylcholine at the cholinergic synapses of nerves causing uncontrolled firing of the synapse [1, 14]. Subclinical effects of chronic OP exposure such as peripheral AChE inhibition may be detected early by biological monitoring tests [1]. Recovery from AChE inhibition can be prolonged due to the binding of OPs and their slow dephosphorylation/breakdown or replacement by fresh AChE enzyme. In red blood cells the recovery half-life is 12–14 days and its time course is related to the recruitment of replacement circulating erythrocytes with a 120 day life cycle [15].

Monitoring exposure to OPs involves the measurement of peripheral cholinesterase enzymes that are inhibited by OPs. These include erythrocyte acetylcholinesterase (AChE) and plasma cholinesterase (PChE). The inhibitory action of OPs on AChE in the nervous system is associated with inhibition of PChE and AChE in blood [16]. Measurement of peripheral ChE enzymes including AChE and PChE has been widely used as a suitable biomarker for potential exposure to OPs and a predictor of adverse effects [17, 18]. Erythrocyte acetylcholinesterase is recommended as a standard laboratory based assessment of OP exposure. It was found that AChE activity is more inhibited by various types of OPs than PChE due to AChE having a slower recovery rate [19–21]. Hence measurement of AChE for identification of chronic exposure and clinical risk assessment is preferred to measurement of PChE [20], which is itself a sensitive index of recent exposure but without a clear link to symptoms [16].

The acute effect of OP exposure is well documented in Australia and overseas for humans, pests and animals [8, 14, 22]. However, the extent to which asymptomatic monitoring is taking place is not well-known or documented in Australia and research overseas is limited. This CROP research builds on the ongoing engagement and farmer friendly health delivery by agricultural health and medicine trained staff at the National Centre for Farmer Health [23, 24]. It uses the proven data collection methods from the award winning Sustainable Farm Families™ program [13], including assessment of health behaviours and self-reported conditions, and adds a sensitive incorporation of reliable field testing procedure for AChE monitoring.

Client engagement and commitment is important due to the method of current colorimetric AChE monitoring [25] and to ensure that an appropriate number of samples are collected to provide an accurate indication of individual AChE activity. It is common for mild to chronic depression of AChE activity to be reported as ‘normal’ due to the wide reference range for AChE activity [26]. It is important, therefore, that farmers and agricultural workers who are routinely using OPs establish their baseline AChE activity and have access to regular AChE activity checks to then monitor changes from baseline.

This study aims to describe the use of clinical point of care sampling to determine the level of chemical exposure associated with the use of OPs by Victorian farmers and their workers. This preliminary study examines the colorimetric methodology of field testing [25] for cholinesterase activity at point of care (POC). Cholinesterase activity is compared amongst farmers over a period of time and also between farmers and non-farmers in South West Victoria. This CROP study will also inform the integration of AChE monitoring into routine point of care health clinics, and provide farming and non-farming people with a comprehensible link between their AChE activity and their household chemical and agrichemical use. It is anticipated that this Cholinesterase Research Outreach Project (CROP) will inform future research examining exposure pathways and patterns in farming communities and the acceptability of POC testing for at risk populations.

Such a monitoring procedure will offer new and valuable data on OP exposure directly to farmers, enabling them to make their own evidence-based decisions to reduce their individual exposures by adopting best practice and adhering to recommended standards of protection.

## Methods

### Study design

This was a prospective cohort study, where farmers and non-famers were compared over time on the levels of AChE at four time points over a 10–12 week period of baseline (first measurement), 3–4 weeks, and 6-7 weeks and at 9-12 weeks. At the same time, change of own AChE levels at each point was compared with the previous readings amongst farmers. The study protocol for this research was published by the authors in 2015 [24].

### Study participants

Study participants (*N* = 55) were residents from South West Victoria, aged between 18 and 75 years, spoke English, and had not had a previous known acute chemical accident. A total of 41 farming (had been farming for more than 5 years) and a convenience sample of 14 non-farming individuals met the inclusion criteria. A screening question was used to determine whether the participant was the primary pesticide user or not. Participants from both groups were not pregnant (self-reported), not suffering from a known chronic disorder, not taking anti-inflammatory drugs and not exercising excessively throughout the study.

### Sampling

Power analysis to detect a difference in cholinesterase activity among farmers before and after exposure and between farming and non-farming groups indicated that a sample size of 30 farmers would be sufficient (power [1-β] = 80%; α =0.05; effect size of one standard deviation obtained from US data cited by EQM Test-Mate [25]. Assuming probable retention rate of at least 85% for a typical 4-month study, the target exposed sample size was 50 participants. This included mixed farming enterprises and dipping and spraying contractors who use OPs. A group of non-farming individuals (*n* = 14) from a rural community of 9000 people was used for comparison.

### Data collection

Consent was obtained from 55 participants, who were recruited via existing contacts and industry groups, letterbox drops and newspaper articles in a number of farming and local newsletters. Trained research staff collected anthropometric and behavioural data. Sociodemographic data was collected directly from the participants including age, gender, country of origin, using the Victorian Department of Health Service Coordination Tools (SCOT) [27]. Data on the type of farming undertaken and residential postcodes were also collected from the participants. Data was collected on the following variables:

- ***Health and behavioural data:*** A structured questionnaire was used to obtain data on current health conditions, smoking and alcohol use. Occurrence of illness or injury experienced within the last three months was measured using a randomised order questionnaire describing 33 symptoms of chemical exposure. Data were also collected on prescribed medication use.
- ***Psychological status data:*** The 10-item Kessler Psychological Distress Scale (K10) questionnaire (a validated short measure of non-specific psychological distress), was used to determine possible distress among the participants in the most recent 4-week period [19].
- ***Physical assessments:*** Weight was measured to the nearest 0.05 kg using electronic scales. This measurement was recorded with the participant wearing light clothing, shoes removed, pockets emptied and prior to breakfast. Height was measured using a portable stadiometer to the nearest 0.1 cm with shoes removed and weight distributed evenly on both feet. Waist circumference was measured to the nearest 0.1 cm at the end of a normal expiration and using a constant tension “Figure Finder Tape Measure”™ [28]. Body Mass Index was calculated using the WHO guidelines [29] for obesity and overweight categories. Body fat percentage was calculated using bioelectric impedance analyser. Blood pressure reading was recorded using a digital blood pressure monitor after allowing the participants to sit comfortably and resting the left arm on a table to keep the blood pressure cuff at about the same height as the heart of the participants. Two separate readings were recorded and an average reading was then calculated. Fasting blood glucose and lipid levels were monitored using capillary blood obtained through finger-prick and strip tests. Respiratory functions were recorded utilizing a Piko6 meter for Fev 1, Fev6 and eyesight was tested using a standard Snellen chart.
- ***Monitoring agrichemical use:*** In addition to completing the 13-question agrichemical-use survey, self-reported chemical use (commercial product name or active ingredient identified) was recorded during each visit. Chemical use was recorded and taken into account during the first AChE measurement of each participant. Participants were not specifically asked to be agrichemical free for a time before commencement of the study. Each participant’s AChE was measured on four occasions (using the EQM Test-Mate Model 400 in AChE mode using the standard methodology [25]. A capillary blood sample of 10 μl was used to measure AChE as units/ml and with activity standardised against whole blood haemoglobin (U/g Hb).

#### Data analyses

All data were managed, analysed and graphed using statistical programs SPSS (IBM Corp. Released 2012 IBM SPSS Statistics for Windows, Version 21.0. Armonk, NY) and Graphpad Prism (version 7 for Windows; GraphPad Software, La Jolia California, USA). Data were examined for normality (Kolmogorov Smirnov test). Following detailed descriptive analyses, inferential analyses were conducted to determine the use of agrichemical products and blood levels of AChE using repeated measures (subjects as own controls) within a General Linear Model. Data were also analysed by one-way ANOVA to compare levels of exposure between farmers and non-farmers. Association between categorical variables was assessed using chi-squared tests and statistical significance was considered at the cut off value of *p* < 0.05.

#### Ethics

The study participants were provided with a plain language statement and informed written consent was obtained. Ethics approval had been granted by the Deakin University Human Research Ethics Committee (HREC 2013–100, dated 18/06/2013).

## Results

Study participants included 41 farmers and 14 non-farmers. The majority of the farmers identified their primary farming type as sheep (51.2%) and cropping (39%). Table 1 shows the health demographics, behaviour and health variables between farmers and non-farmers. The majority of the participants were male and belonged to the age group of 45–64 years. Although there was no significant difference in average age between farmers and non-farmers, farmer participants were more likely to be male. Irrespective of farming status, most of the participants perceived their health as ‘good’ to ‘excellent’. Very few farmers (2.4%), categorised their health as ‘excellent’, in comparison to non-farmers (42.9%) which is statistically significant. Survey responses showed more farmers experienced ‘moderate’ to ‘very high’ distress indicated in the Kessler 10 scale compared to non-farmers (31.7% vs. 14.3%), although the difference was not statistically significant due to the small sample size.

**Table 1:** Demographics, behaviour and health status of farming and non-farming participants

Variables	Farmers, n(%)	Non-farmers, n(%)
Total participants	41 (100)	14 (100)
Age (years)		
Mean (±SD)	45 (10.1)	43 (10.2)
Age groups		
25–44 years	19 (46.3)	6 (42.9)
45–64 years	19 (46.3)	7 (50.0)
65+ years	3 (7.3)	1 (7.1)
Gender		
Male	38 (92.7)	9 (64.3)
Female	3 (7.3)	5 (35.7)
Overall health		
Perceived health status		
Excellent	2 (4.8)	6 (42.9)
Very good	27 (65.9)	7 (50.0)
Good	11 (26.8)	1 (7.1)
Fair	1 (2.4)	0 (0)
Bodily pain in last 4 weeks	*(n = 38)*	*(n = 14)*
None	0 (0)	1 (7.1)
Very little	18 (47.3)	4 (28.6)
Some	17 (44.7)	9 (64.3)
Severe	3 (7.8)	0 (0)
Wellbeing (K10 score range)		
Low (10–15)	28 (68.3)	12 (85.7)
Moderate (16–21)	12 (29.3)	1 (7.1)
High (22–29)	0 (0)	1 (7.1)
Very high (30+)	1 (2.4)	0 (0)
BMI categories		
Underweight (≤19)	0 (0)	1 (7.1)
Acceptable (20–24.99)	14 (34.1)	6 (42.9)
Overweight (25–29.99)	21 (51.2)	5 (35.7)
Obese (30+)	6 (14.6)	2 (14.3)
Cholesterol risk		
Low (< 5.5)	31 (75.6)	9 (64.3)
High (≥5.5)	9 (22.0)	5 (35.7)
Waist measurement risk level		
Female low risk (< 88 cm)	3 (7.3)	4 (28.6)
Female high risk (≥88 cm)	0 (0)	1 (7.1)
Male low risk (< 102 cm)	25 (61.0)	6 (42.9)
Male high risk (≥102 cm)	12 (29.3)	3 (21.4)
Blood Glucose risk level		
Low (Fasting < 5.5)	27 (67.5)	7 (50)
High (Fasting ≥5.5)	12 (30)	7 (50)
Low (non-fasting < 6.5)	2 (2.5)	0 (0)
Respiratory Assessment	*(n = 38)*	*(n = 12)*
Red (< 0.65)	0 (0)	0 (0)
Yellow (0.65–0.75)	2 (5.2)	1 (8.3)
Green (≥0.75)	36 (94.7)	11 (91.6)
Smoking		
Current smoker	4 (7.3)	0 (0)
Ex-smoker	11 (26.8)	4 (28.6)
Never smoker	26 (63.4)	10 (71.4)
Frequency of alcohol containing drink intake	*(n = 40)*	*(n = 14)*
Never	2 (5.0)	1 (7.1)
Monthly	3 (7.5)	3 (21.4)
Once a week	6 (15)	1 (7.1)
2 to 4 times per week	20 (50)	7 (50.0)
5+ per week	9 (22.5)	2 (14.3)
Short term risky alcohol use	*(n = 39)*	*(n = 13)*
Never	8 (20.5)	6 (46.1)
Monthly	12 (30.7)	5 (38.4)
Once a week	12 (30.7)	1 (7.6)
2 to 4 times per week	7 (17.9)	0 (0)
5+ per week	0 (0)	1 (7.6)

A large number of the farmer participants reported that they both use and self-apply agrichemicals, 70.7% self-apply insecticides and 82.9% self-apply herbicides, this is significantly different from the non-farmer responses Pearson Chi square (Σ^2^ (df 5, *N* = 55), *p* < 0.001) (Table 2). Self-reported results showed that the majority of the farmers (92.7%) used some form of personal protective equipment (PPE) when mixing and applying chemicals. The use of PPE among the non-farming participants was very low (14%). Gloves were the most frequently used form of PPE with 58.5% of farmers ‘always’ using gloves when loading, mixing or applying chemicals. Face protection was not widely used by the farmer participants, and 51% of all participants reported ‘never’ using face protection when working with agrichemicals.

**Table 2:** Self-reported use of chemicals and personal protective equipment (PPE) in farming and non-farming participants (*X 2 (df 5, N = 55), *p* < 0.001)

Variables	Farmers, n (%)	Non-farmers, n (%)
Total participants	41	14
Agrichemicals Used or Self-Applied *Insecticides*		
Not Used	6 (14.6)	2 (14.2)
Used but not self-applied	5 (12.1)	0 (0)
Used and self-applied	*29 (70.7)	1 (7.1)
Not Applicable	0 (0)	11 (78.5)
No Response	1 (2.4)	0 (0)
*Herbicides*		
Not Used	2 (4.8)	1 (7.1)
Used but not self-applied	4 (9.7)	0 (0)
Used and self-applied	*34 (82.9)	2 (14.2)
Not Applicable	0 (0)	11 (78.5)
No Response	1 (2.4)	
PPE Frequency of use: *Gloves*		
Always	**24 (58.5)**	1 (7.1)
Sometimes	10 (24.4)	1 (7.1)
Never	4 (9.7)	0 (0)
Not Applicable	3 (7.3)	12 (85.7)
*Face Protection*		
Always	2 (4.8)	0 (0)
Sometimes	15 (36.5)	0 (0)
Never	18 (43.9)	1 (7.1)
Not Applicable	6 (14.6)	13 (92.9)
*Overalls/Coveralls*		
Always	5 (12.2)	0 (0)
Sometimes	14 (34.1)	1 (7.1)
Never	17 (41.5)	0 (0)
Not Applicable	5 (12.2)	13 (92.9)
*Mask/Respirator*		
Always	4 (9.8)	1 (7.1)
Sometimes	22 (53.6)	1 (7.1)
Never	11 (26.8)	0 (0)
Not Applicable	4 (9.8)	12 (85.7)
Stirring/Mixing Agrichemicals		
Hand/arm	1 (2.4)	0 (0)
Auto stirrer	14 (34.1)	0 (0)
Stick or paddle	**15 (36.6)**	2 (14.3)
Other	6 (14.6)	0 (0)
Not Applicable	5 (12.2)	12 (85.7)

Retention of participants in the study was high, with 95% of farmers and 100% of non- farmers returning for all follow up AChE readings (four readings in total). The use of agrichemicals prior to and/or during the study was found to be 95% (39 out of 41) among the farming participants, 14.6% of which included an organophosphate (AChE inhibiting) compound (Methidathion, Dimethoate, and Omethoate). No other anticholinesterase compounds/groups i.e. Carbamates, were reported by farmers in this study. The most commonly other used chemicals by farming participants in the weeks leading up to the study were Glyphosate and MCPA both at 14.6%. Glyphosate was also the most commonly used chemical in the non-farmers.

**Fig. 1:**
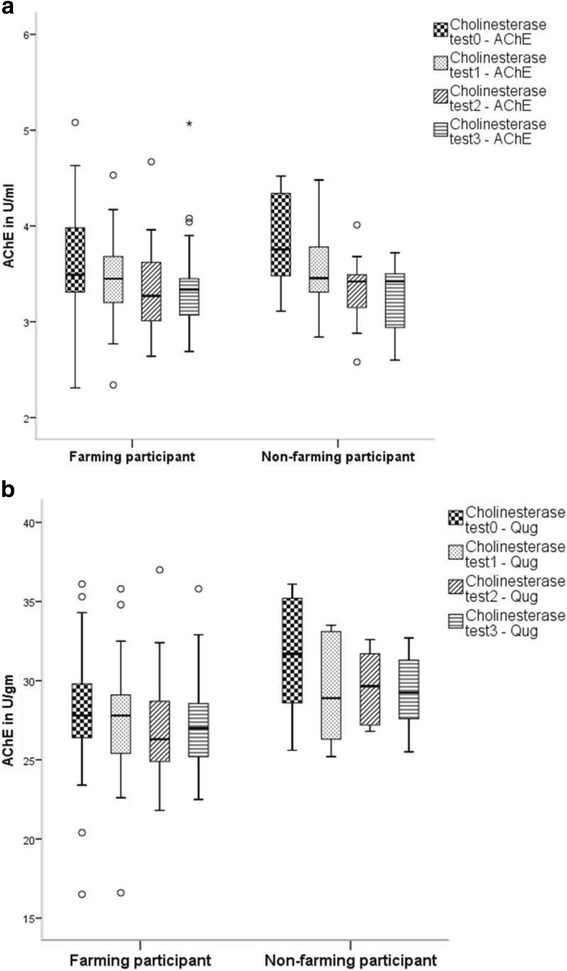
Frequency distribution for AChE activity in farming (*n* = 41) and non-farming participants (*n* = 14); **a)** AChE in U/ml and **b)** AChE in U/g; (Baseline: Cholinesterase test 0, At 3–4 week intervals: Cholinesterase test 1, At 6-weeks: Cholinesterase test 2, At 9-weeks: Cholinesterase test 3)

Despite differences in reported chemical usage and use of PPE, data collected from farmers and non-farmers showed no significant difference in average AChE activity between farming and non-farming participants (one-way ANOVA *p* > 0.05). Figure 1 shows the distributions of AChE for both farming and non-farming groups.

**Fig. 2:**
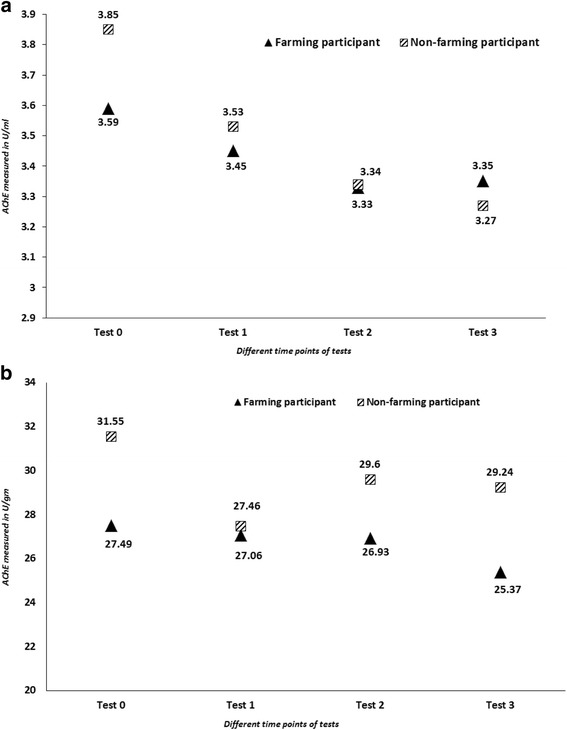
Change of mean AChE activity in farming (n = 41) and non-farming participants (*n* = 14); **a)** AChE in U/ml and **b)** AChE in U/g

A one-way ANOVA was conducted to compare the effect reported personal use of agricultural chemicals on farm had on levels of AChE at baseline or any of the follow up periods T0 F(1, 53) = 0.688, T1 F(1,53) = 0.239, T2 F(1,53) = 1.25, T3 F(1,53) = 1.28 (*p* > 0.05). There was also no significant difference whether the participants were farmer or non-farmer T0 F(1, 53) = 2.53, T1 F(1,53) = .392, T2 F(1,53) = 0.018, T3 F(1,53) = 0.391 (*p* > 0.05). However, a repeated measures ANOVA with a Greenhouse-Geisser correction among the total participants determined that the mean activity of AChE was significantly lower within follow up periods [F (2.633, 139.539) = 14.967, *p* < 0.0005] (Fig. 2). Post hoc tests using the Bonferroni correction revealed that there was a statistically significant reduction in AChE activity comparing the baseline value (3.66 ± 0.54 U/ml) with 3-weeks of monitoring (3.47 ± 0.43 U/ml, *p* = 0.013), 6-weeks of monitoring (3.32 ± 0.39 U/ml, p < 0.0005), and 9-weeks of monitoring (3.33 ± 0.41 U/ml, p < 0.0005). Further analyses of the time period indicate that there was a significant reduction of AChE between the follow up sessions at 3-weeks and 6-weeks (*p* = 0.015), but not between the 6-weeks and 9-weeks (*p* > 0.05) among the total study participants.

When the same analyses were conducted individually for farmers and non-farmers, the significant reduction was also observed at 3-weeks and 6-weeks period, but not between the 6-weeks and 9-weeks period. When adjusted for haemoglobin (Hb), there were no participants that displayed a reduction in AChE activity of greater than 25%, all participants (farmer and non-farmer) measured only slight AChE inhibition between T0 and all other time points.

## Discussion

This paper describes the measurement of AChE enzyme activity on control (non-farming) participants and farming participants and the value of incorporation of these measurements into routine Point of Care (POC) health clinics. Organophosphates are still used widely in South West Victoria and globally and the actions and attitudes of farmers and workers using these pesticides are vital to farmer wellbeing and that of their families. When examining enzyme activity as an index of chemical exposure and potential effect, it is important to establish the criteria determining whether there has been an effect. In biological monitoring there are several ways to evaluate data;

*Examination of grouped data* from farmers, farming spray contractors, non-farming rural residents and non-farming town residents provides a blunt instrument for drawing associations between general farming practices and use of chemicals, broad environmental exposure and effect.

*Comparison of individual data* against standards or guidelines outlining acceptable levels of the measured index, suggest unacceptable (or clinically relevant) levels of exposure have taken place. It is generally accepted that depression of AChE by 70% or more may be associated with the appearance of acute symptoms. The question remains as to what value represents 100% activity. As was the case with this sample group, cholinesterase activities are normally distributed within populations, and those with normally lower cholinesterase activity may be misidentified as chemically-exposed, whereas those with higher cholinesterase activity may be identified as unexposed, even where there may be significant inhibition relative to an even higher ‘normal’ cholinesterase. A comparison of country specific data may be preferable since the general Australian population may differ significantly from the US data set used by EQM. A recent study undertaken by Suratman et al. reported mean erythrocyte AChE for 27 unexposed blood donors from South Australia was 3.24 U/mL (standard deviation ±0.40) and 29.6 u/g Hb (SD ±3.0) [2]. This suggests that using Australia specific data with concomitantly lower population background values, fewer workers are now identified as possibly exposed or poisoned. This approach, however, is still flawed as it depends upon individual data comparisons with population distributions. Low AChE does not necessarily mean inhibited AChE.

Comparison of individual data with established individual baseline measures (ideally preceding exposure or employment) allows for individual trends in measurements to be related to recent occupational practices or regional factors affecting more widespread chemical exposure [30]. There can also be difficulties, in establishing ‘true’ or stable baseline estimates for individuals, especially where farmers may handle anticholinesterase insecticides on an ongoing or year-round basis, and short breaks or vacations may not be sufficient to allow AChE to return to baseline levels. Similarly, while some farmers may suspend insecticide use, neighbours may continue using them. This increases the possibility of more persistent general community exposure to insecticides which may contribute to a low level depression of cholinesterase, upon which the effects of further occupational or environmental exposures may be superimposed. It is important that test results be interpreted in conjunction with reported use of personal protective equipment (PPE).

This research has revealed that a reasonably high number of farming participants (92.7%) reported use of ‘some form’ of PPE when mixing and applying chemicals, with gloves being the most widely adopted form of protection and face protection the least utilised (51% reported never using face protection). A report by Franklin et al. (2015), suggests that understanding how individuals perceive threats, make decisions and adopt new ideas, provides insight into the ways that barriers to PPE use can be identified, and intervention can be designed and evaluated. Work on occupational noise exposures by Williams reported that whilst conducting a test and providing participants with a result is helpful, it is not enough to incite practice change [31].

A later study undertaken by Brumby et al. on noise induced hearing loss on farms showed that provision of accurate individual hearing loss measurement in combination with noise levels on farm and education has proven an effective method for improving farm safety practices and reducing individual noise exposure [32]. These contributions are important when considering farmers exposures to OP’s and possible future interventions to adopt new safety practices.

Point of Care (POC) testing is covered by ISO 22870 Point of care testing –requirements for quality and competence, but this refers mainly to distributed locations, such as GP clinics and pharmacies, rural health clinics, nursing homes, sports medicine clinics and extends to workplace drug screening [33]. There is little guidance on the application of POC testing in the circumstances described here in relation to OP exposure. In particular, the timing of further tests should be based on the nature of the work and previous test results. According to Safework Australia 2013 guidelines a worker having greater than 20% inhibition from baseline values should be retested within 12 weeks [34].

Workers must be advised of their results (that is, percentage depression of cholinesterase in relation to chemical use and possible exposure) to permit them to understand the basis for any restrictions or changes to work practices. Participants in this study were provided with a summary of their health measurements and AChE results at the conclusion of the study along with education on reduction of exposures. This generated considerable interest amongst participants with many recognising fluctuations in AChE measures may reflect personal exposure. Whist this is positive whether participants have changed their behaviours and safety practices to reduce exposure is yet to be quantified. The retention rate of 93% of participants returning for 4 consecutive monitoring sessions indicated that farmers involved in this study remained engaged and were concerned about possible exposure to organophosphates. In spite of these individual fluctuations in AChE levels, the current data suggests there was no significant difference in observed average AChE activity between farming and non-farming participant groups, although the sample size was relatively small. This is consistent however, with a 2012 study by Pasiani et al. of Brazillian farmers which found significant differences between the mean AChE activities of the farmer group during both non-exposure and exposure periods [35].

This result reflects the responses provided by farming participants when the type of chemicals used in the weeks prior to and during the study. Whilst only 14.6% of participants (farming only) reported using organophosphate pesticides throughout the study, it was the equal top chemical group used along with the single herbicides; glyphosate and MCPA. This is reflective of the variety of agrichemical use within different farming enterprises (cropping (29.1%), sheep (40%) or cattle/dairy (1.8%)). Given the short timeframe in which the research was undertaken the chemicals reported may not have been a true representation of the variety of agrichemicals used by this group of farmers. Further work is required to better determine the true extent of anticholinesterase chemical use throughout western Victoria.

It is important that producers who are routinely using OPs establish their baseline AChE activity and have access to regular AChE activity checks for comparison with baseline. A 2001 study by Dyer et al. suggests that differences in patterns of availability and domestic use of anticholinesterase chemicals including chlorpyrifos and similar agents (that may be banned elsewhere), by nominally ‘control’ populations may affect peripheral cholinesterases and may influence our adoption of standard or guideline values. In addition, chlorpyrifos may affect plasma cholinesterase whereas other agrichemicals such as dimethoate and diazinon more potently effect erythrocyte cholinesterase [18].

Participants were provided with counselling regarding their health and behaviour measurements and AChE levels in accordance with Australian AgriSafe™ guidelines using US normal data. It would be preferable to provide country or region specific comparisons to allow workers a better understanding of their own chemical exposure.

Incorporation of POC testing into health clinics and emergency care is highly dependent on health professional’s attitude, skills and knowledge and availability of testing equipment. The competency of health professionals is crucial to the retention of farming participants who require follow up cholinesterase monitoring. Rajapakse et al. 2014 suggest that greater experience by health professionals in seeing AChE test results upon an acute poisoning presentation is associated with increased knowledge [36]. The results of this study further prepare health providers for integration of appropriate cholinesterase measurement into POC health check procedure. The critical window for exposure to toxicants may occur years before the onset of neurological symptoms [10]. This work further highlights pesticide exposure as a risk for farmers and their families, leading to work that will permit quantitation of environmental exposure and early detection in the workplace and homes of farming and non-farming individuals. These types of data may also be included in epidemiological studies of chronic diseases. Research has also shown links between cholinesterase activity and vascular complications in diabetic patients [37]. As diabetes is a disease of increasing concern to the farming and rural population in Australia, continuing to develop a database of health and lifestyle data which includes chemical usage and cholinesterase activity would be valuable to further understanding and addressing complex Australian public health comorbidities [38]. It is now known that consideration of both time and frequency of interventions is vital to ensure behaviour change is successful. Ongoing interaction in some form is required to further embed changes in attitudes, practices and behaviour [32].

## Conclusions

For any effective preventive program to reduce exposures farmer engagement is crucial. This pilot study reports on the successful integration of POC cholinesterase monitoring into rural health clinics where OPS are used. It also provides an evidence base which can further quantify pesticide exposure both on the farm and in the home of farming families. Given a reasonable estimate of baseline AChE is available, the routine monitoring of AChE may allow for early recognition of chronic low-level exposure to OPs when they are in use by farmers. It is suggested that non-farmers may also be exposed but are less likely to regularly monitor their chemical exposure and use PPE when using chemicals. Providing access to this type of biological monitoring at POC, represents a critical opportunity to repeatedly engage farmers in understanding decisions that can protect and improve their health and minimise their agrichemical exposure. Further research is required to evaluate if participation in agrichemical monitoring research and farmer health clinics, reduces exposure through changes in farmers attitudes and safety practices.

### Limitations

Results suggest that there are differences in the mean AChE levels between various sampling times. This may be a reflection of the differences in enterprise type between individuals and the varied use of chemical during different times of the year. Chemical use was only recorded at the commencement of the study and participants were not specifically asked to be agrichemical free for a time before commencement of the study. This limitation has may have restricted the accuracy with which correlations could be made between chemical use and differences observed in AChE levels between time points (Fig. 2). However absences from work for holidays by were accompanied by individual increases in cholinesterase. The self-reported usage of chemicals by non-farming participants accounts only for perceived exposure to chemicals. Further work is required to more accurately determine the true exposure and chemical usage and low-level exposures of farming and non-farming populations throughout the duration of a research period.

Future research should aim to quantify pesticide exposure on the farm and in the homes of farming and non-farming families, using reliable estimates of ChE activity. Further work should also highlight the importance of providing a safe work environment for farming communities.

## Abbreviations

AChE: Erythrocyte Acetylcholinesterase
BMI: Body Mass Index
CROP: Cholinesterase Research Outreach Project
HDL: High Density Lipoproteins
K10: Kessler 10
LDL: Low Density Lipoproteins
NCFH: National Centre for Farmer Health
OP: Organophosphate.
PChE: Plasma Cholinesterase
POC: Point of Care
SCOT: Service Coordination Tools
